# Use of Artificial Intelligence Model Associated with Masson’s Trichrome Staining as a Predictor of Muscle Invasion in Bladder Cancer

**DOI:** 10.3390/ijms27052237

**Published:** 2026-02-27

**Authors:** Diego Parrao, Hector Gallegos, Karin Ruz, Román Lay, Catalina Saavedra, Renata Guerrero, Matías Larrañaga, Carolina B. Lindsay, Juan Cristóbal Bravo

**Affiliations:** 1 School of Medicine, Universidad de O’Higgins, Rancagua 2820000, Chile; diego.parrao@pregrado.uoh.cl (D.P.); roman.lay@pregrado.uoh.cl (R.L.); renata.guerrero@pregrado.uoh.cl (R.G.); 2 Department of Urology, Hospital Sótero del Rio, Santiago 8207257, Chile; 3 Department of Pathology, Hospital Dr. Franco Ravera Zunino, Rancagua 2820000, Chile; 4 Department of Urology, Hospital Dr. Franco Ravera Zunino, Rancagua 2820000, Chile; 5 Research Department, Hospital Dr. Franco Ravera Zunino, Rancagua 2820000, Chile

**Keywords:** Masson’s trichrome staining, bladder cancer, muscle invasive, convolutional neural network

## Abstract

Bladder cancer (BC) is the most common malignancy of the urinary tract. Approximately 75% of cases are non-muscle-invasive BC (NMIBC), while muscle-invasive BC (MIBC) and advanced tumors account for most cancer-specific mortality. Accurate assessment of tumor invasion is essential, as staging variability may lead to inappropriate treatments. Tumor invasion involves several mechanisms including extracellular matrix (ECM) remodeling mediated by metalloproteinases, angiogenesis, and cell adhesions. Masson’s trichrome staining (MTS) provides relevant information on ECM composition. This study evaluated the application of machine learning to MTS-stained bladder biopsies to predict muscle invasion. A retrospective analysis of bladder biopsy images obtained from transurethral resections and cystectomies (2022–2024). A total of 702 histological images were analyzed. A convolutional neural network (CNN) was trained to classify tumors as MIBC or NMIBC and model outputs were correlated with clinical variables. The CNN achieved an accuracy of 95.2% in the training set and 90.1% in validation. Model-derived probabilities were significantly associated with tumor grade, lesion size, and muscle invasion. Logistic regression demonstrated a strong association with invasive disease (OR = 0.07, *p* = 0.017). CNN-based analysis of MTS-stained bladder biopsy images enable accurate prediction of muscle invasion, with potential to improve diagnostic precision.

## 1. Introduction

Bladder cancer (BC) is the seventh most commonly diagnosed cancer in males, with 600,000 patients being diagnosed in 2020 with bladder cancer worldwide [1]. In non-muscle-invasive BC (NMIBC), approximately 75% of patients present with disease confined to the mucosa (stage Ta), carcinoma in situ (CIS), or submucosa (stage T1), although most of the cancer-specific mortality is associated with T2–T4 tumors, classified as muscle-invasive BC (MIBC). NMIBC can progress to muscle-invasive or metastatic disease in approximately 15% of patients; however, high-grade NMIBC cases can reach a 45% rate of MI progression within 5 years, associated with a 50% 5-year survival rate, despite aggressive treatments such as radical cystectomy (RC) [1,2,3,4,5]. Nowadays, further approaches are needed to not only refine risk stratification of muscle invasion but also to guide personalized therapeutic strategies according to it and, therefore, improve overall outcomes for BC patients, addressing a critical gap in uro-oncological care.

The standard method used to determine stage of neoplasia in BC is transurethral resection of the bladder tumor (TURBT); this procedure provides treatment for patients with NMIBC and staging information for those with MIBC through histopathological biopsy analyses; however, the inter-observer difference between pathologists can vary between 27% and 74% [6,7,8,9,10]. In BC, staging of the primary lesion by TURBT is fundamental to a rational therapeutic approach; the absence of detrusor muscle and high-risk tumors in the TURBT derived specimen is associated with a high risk of residual disease, early recurrence, and tumor understaging. Therefore, in these cases, a second TURBT should be performed to achieve a precise diagnosis, exposing the patient to a new invasive procedure and its associated complications [11,12,13]. Although much work has been done in the area of detecting early cancer, only few studies have focused on accurately identifying muscle invasion in BC cases [14,15,16,17]. The search for new methods that could improve the presence of MI in BC patients is necessary to improve the concordance between pathologists and diminish the amount of second resection needed that could lead to less complications, better quality of life, and less economic burden.

Muscle invasion requires more aggressive therapeutic approaches as it involves a complex interaction between tumor cells and their microenvironment, overcoming surrounding matrix and tissue barriers and colonizing adjacent structures through migration. In BC, this phenomenon represents not only a morphological change but also molecular reprogramming orchestrated by a network of biological signals that includes modifications in gene expression, activation of oncogenic signaling pathways, and remodeling of the extracellular microenvironment. Regarding the tumor microenvironment, the extracellular matrix (ECM) is a dynamic and highly structured component that modulates not only tissue support but also cancer cell behavior, influencing invasion, differentiation, and angiogenesis [18,19]. However, tumors can often also induce changes in the ECM of the connective tissue, including alterations in collagen density, cross-linking, and degradation of it. ECM remodeling, often promoted by the secretion of matrix metalloproteinases (MMPs) by tumor and stromal cells, facilitates cell migration by generating pathways through which tumor cells can invade surrounding tissues [20,21]. The interaction between tumor cells and an unstructured ECM can favor muscle invasion, a critical marker of cancer progression and prognosis [22]. The study of the state of the ECM in this context could allow us to evaluate its contribution to tumor migration and identify patterns that help in detecting muscle invasion.

Imaging techniques and molecular assays targeting these ECM changes can provide valuable insight into the tumor microenvironment dynamics, aiding in the early detection of muscle invasion. MTS is a well-known histological technique widely used to evaluate the composition and organization of the ECM in tissues, due to its ability to differentiate collagen, muscle, and cellular cytoplasm [23,24]. In the context of BC, this stain could offer significant diagnostic value by allowing clear visualization of the tumor cells and their environment, including potential muscle invasion associated with the ECM state. The ability to accurately identify the presence of unstructured or reorganized collagen fibers and changes in the integrity of the muscularis propria could be useful in determining patterns associated with cell migration, facilitating the correlation between histological findings and underlying oncological mechanisms. However, traditional diagnostic methods including histopathological evaluation using staining techniques can be limited by subjectivity and inter-observer variability [6,7,8,9,10].

Nowadays, machine learning (ML) is revolutionizing the field of medical diagnostics by enabling the analysis of complex datasets with unprecedented accuracy and efficiency [25,26,27]. In the context of diagnosing MIBC, ML could offer a powerful tool to complement traditional histopathological techniques through algorithms capable of detecting patterns and features within biopsy images, enhancing diagnostic precision and reducing inter-observer variability inherent to human assessments. The application of ML to analyze bladder biopsies stained with Masson’s trichrome staining represents an impactful step towards integrating computational intelligence with clinical pathology, with the potential to significantly improve patient diagnosis and outcomes.

By integrating ML into the MTS analysis of biopsy samples, this study seeks to improve the diagnostic accuracy of muscle invasion in BC. This allows for a more standardized and reproducible assessment of tumor characteristics, ultimately improving clinical decision-making. Given the wide inter-observer variability that often necessitates a second TURBT, we believe that an artificial intelligence model could offer greater diagnostic accuracy. Consequently, this could reduce overtreatment, undertreatment, and the need for repeat procedures, which is what we are trying to develop with this model and analysis.

## 2. Results

**Figure 1:**
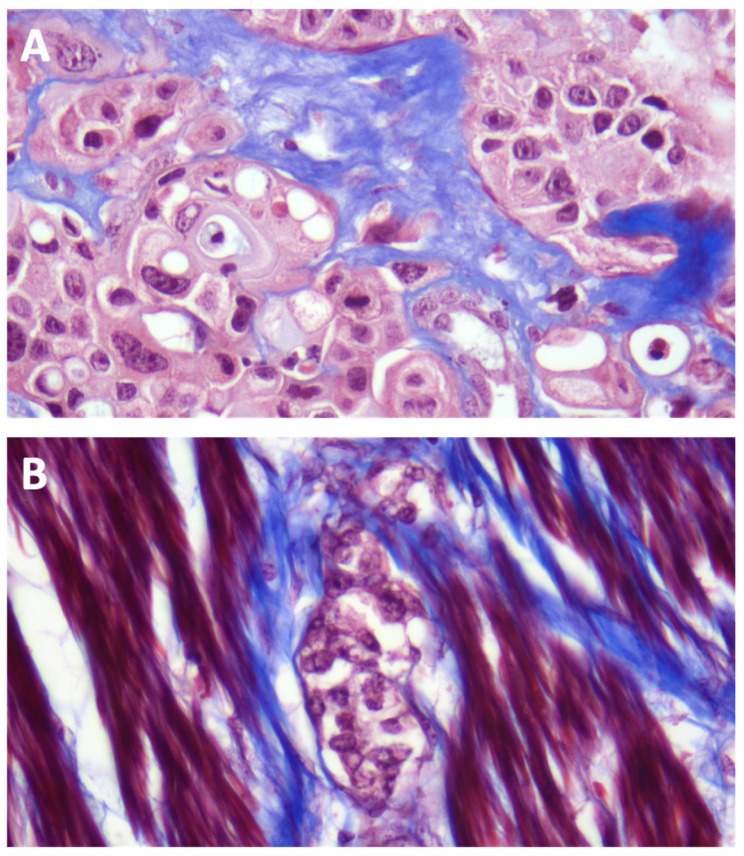
Representative images from Masson’s trichrome-stained samples (NMI and MI). (**A**) Masson’s trichrome stain (400×). High-grade urothelial carcinoma infiltrating the lamina propria. Stromal collagen fibers stained blue and neoplastic urothelial cells stained pink. (**B**) Masson’s trichrome stain (400×). High-grade urothelial carcinoma infiltrating the muscularis propria. Stromal collagen fibers stained blue, muscularis propria red/pink, and neoplastic urothelial cells pink.

A total of 32 patients with BC were included; 702 images were taken and submitted to the artificial intelligence model (Figure 1), of which 22 were NMIBC and 10 MIBC: 12 were Ta, 11 T1, 7 T2, and 2 T3; mean age was 73 years for NMIBC, while for MIBC it was 66.3, the probability of detecting muscle invasion was 0.487 and 0.998 for NMIBC and MIBC images, respectively (*p* = 0.001) (Table 1).

Our model achieved an accuracy of 95% for the training process and up to 90.1% in the validation process. The probability generated by the model was correlated with tumor grade, tumor size, tumor weight, which had a clinically significant association (*p* = 0.005, 0.017 and 0.01, respectively) (Table 2). In addition, sensitivity and specificity were calculated for diagnosis by AUC of ROC curves obtaining a sensitivity of 90%, specificity of 91%, a positive predictive value of 82%, and a negative predictive value of 95% (Table 3).

In addition, an adjustment was performed by a multivariate analysis with other clinical factors such as size, grade, weight, and probability of the model. The probability of detecting muscle invasion from the MTS biopsy images remains statistically significant (*p* = 0.019). No significant differences were observed when size (*p* = 0.296), grade (*p* = 0.51), and weight (*p* = 0.801) were compared (Table 4).

## 3. Discussion

The development of this model is set against a backdrop of rapid advancements in the implementation of artificial intelligence within the medical domain. Recently, AI has shown significant progress in research and clinical diagnostics, highlighted by milestones such as the AlphaFold model for structural biology [28] and the integration of large language models (LLMs) into clinical workflows [29]. The present study represents a specific application of this revolution within computational pathology: the transformation of complex microscopic image patterns into clinically meaningful diagnostic indicators.

Artificial intelligence (AI) has consolidated its role as a supportive tool at various levels of the diagnostic and therapeutic process for BC, optimizing procedures such as cystoscopy, urinary cytology, and prediction of clinical outcomes. AI has been utilized for the automated detection of tumor lesions in endoscopic images, increasing diagnostic sensitivity compared to conventional evaluation [15]. Furthermore, models capable of predicting tumor recurrence and progression have been developed through the analysis of clinical and imaging data, giving strength to risk stratification [15]. Also, the use of AI in bladder biopsies has been investigated to improve the accuracy of the diagnosis and reduce inter-operator variability.

**Figure 2:**
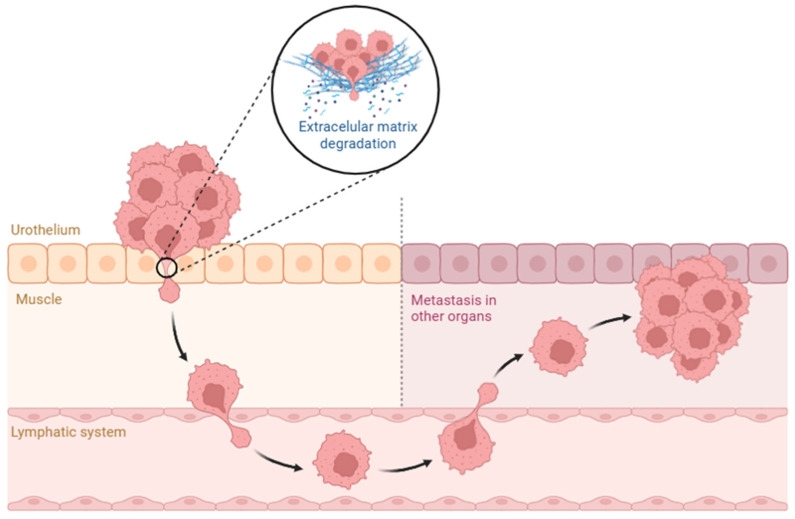
Schematic representation of bladder cancer invasion and metastatic dissemination. Tumor cells originating in the urothelium progressively invade the underlying muscle layer through degradation of the extracellular matrix (ECM). Once tumor cells breach the basement membrane, they can enter the lymphatic system, facilitating their dissemination to distant sites. Migrating cancer cells eventually colonize other organs, where they establish secondary tumors and promote metastatic growth.

The role of ECM remodeling and connective tissue status in tumor progression and muscle invasion in BC is still under investigation. Through the use of MTS, we demonstrated its utility not only in detecting muscle invasion but also in providing insights into the structural alterations in the ECM associated with tumor cell invasion. These observations underscore the interplay between tumor cells and their microenvironment, where ECM degradation and reorganization facilitate invasive behavior and, therefore, serve as an indicator of muscle invasion (Figure 2). Our work showed the ability of the MTS technique to distinguish key histological features of the ECM in tumor biopsies, which, in addition to ML algorithms, makes it a valuable tool in clinical diagnostics, particularly in identifying muscle invasion. Our model achieved an accuracy of 90.1% in the validation process and reached 95.2% in the training to differentiate NMIBC from MIBC, reflecting significant differences in tumor grade, tumor weight, and tumor size.

The present study demonstrated the robust capability of the MTS technique to distinguish key histological features of the ECM in tumor biopsies, which integrated with ML algorithms, establishing it as a valuable clinical diagnostic tool, specifically for identifying muscle invasion. Our model achieved an accuracy of 90.1% in the validation process and an accuracy of 95.2% in training for differentiating NMIBC from MIBC. This accuracies are considered high compared to other histopathological-based AI diagnostic models and could be helpful in complex cases diagnoses. Some studies, such as those conducted by Khosravi et al. [30] and Yin et al. [31], demonstrated the potential of CNNs in bladder cancer staging; however, these HE-based approaches frequently encounter a diagnostic bottleneck at the T1–T2 interface. While some of these studies report accuracies near 92% in differentiating early stages (Ta vs. T1) through manual feature selection [31], fully automated CNN models trained on HE sections typically achieve significantly lower accuracies of approximately 84% when tasked with detecting muscle invasion. Other findings by Fahoum et al. [32] underscore this challenge, reporting an analytical specificity of only 46% when identifying muscularis propria invasion using CNNs on HE patches, primarily due to the algorithm misclassifying vascular smooth muscle as detrusor muscle.

This inherent limitation of HE staining lies in the overlapping eosinophilic appearance (similar pink tones) of both the stromal collagen and the muscularis propria, which can induce algorithmic misclassifications due to the inability to precisely delineate the extracellular matrix (ECM) during micro-invasion. Conversely, our application of MTS effectively bypasses this limitation by providing a clear colorimetric distinction, staining collagen blue and muscle fibers red [33,34]. This high contrast allows the CNN to extract structural features of the ECM architecture more precisely, which is fundamental, given that malignant cells frequently disrupt and reorganize collagen fibers to facilitate local invasion and dissemination, and, consequently, dense or aberrantly organized collagen patterns in peritumoral regions are considered predictive of deeper muscular involvement and disease progression [35,36]. Consequently, the MTS-based model serves as a diagnostic tool capable of surpassing the sensitivity of conventional staining by objectively identifying the transition toward MIBC with a superior validated accuracy of 90.1%.

AI-driven image analysis has the potential to significantly reduce variability in cancer muscle invasion diagnosis and improve patient outcomes in oncology. With the support of advanced algorithms, AI can enhance the precision of diagnostic imaging, which is crucial for accurately assessing muscle invasiveness in cancers such as bladder cancer [37]. AI algorithms can achieve comparable or even superior performance to human experts in detecting and classifying cancer, thereby reducing diagnostic variability [38,39]. In this context, inter-observer variability between pathologists in identifying muscle invasion varies between 27% and up to 74%, depending on the studies, of which up to 28% of the cases may even require some change in their treatment [6,7,8,9,10]. These differences in the pathological interpretation of TURBT specimens include the presence of technical artifacts, distinction between muscularis mucosal and muscularis propria, absence of muscularis propria, distinction between stromal desmoplasia and muscular invasion, tangential sectioning, and lack of spatial orientation caused by random inclusion of bladder tissue, which leads to inter-observer variability as well as risk of under- or overstaging, which may lead to inappropriate therapeutic decisions [7,8,9].

For the management of NMIBC, TURBT is a procedure with low associated morbidity. Although life threatening events are rare, complications include tumor cell dissemination, hematuria, bladder spasms, urinary retention, urinary tract infection, and bladder perforation. Complication rates reported in different studies range from 4% to 6%, with urinary tract infection and hematuria being the most common, while bladder perforation requiring surgical repair occurs in 0.5% to 8.3% of complications [40,41,42]. On the other hand, for patients with MIBC, the standard treatment is radical cystectomy; however, despite management, the overall 5-year survival rate is around 60%, and the decision to perform surgery must be weighed against the risks, the impact on quality of life, and the patient’s preferences [43,44]. Early complications, such as infectious, genitourinary, gastrointestinal, and wound-related complications, occur in up to 58% of patients with MIBC, and perioperative mortality has been reported by 3.2% at 30 days and 5.2% at 90 days. Additionally, patients’ independence, sexual, social, and psychological function, body image, and economic status are impaired, significantly impacting their quality of life [4,45,46,47,48]. In our center, during 2024, 64 TURBTs were performed, 12 of which were re-TURBTs (18%). Considering the model’s precision of 91.2%, 11 of these 12 re-TURBT cases could potentially have been avoided. This represents a reduction in hospital burden, equivalent to 11 additional operating room slots for other procedures and approximately 20 h of urologist availability for other tasks, such as outpatient consultations. Such efficiency is vital for public health systems operating with limited resources.

It was observed in a study, which built a model to evaluate the economic impact of bladder cancer in the USA, that the average cost per patient for NMIBC was $31,375 USD and $56,247 USD for MIBC [49]; however, these costs could vary depending on the therapy used; a high-risk NMIBC treated with BCG could result in costs exceeding $200,000 USD after 5 years of management, and an MIBC on trimodality therapy can exceed $200,000 USD for each year of treatment [50]. All these differences in morbimortality and costs between the treatment in NMI and MI disease highlight the importance of properly distinguishing the level of invasion.

This instrument could also reduce the need for re-TURBT when there is diagnostic uncertainty, which, in the literature, is described as varying from 17% to 71% [16,17,51]. This could also reduce the costs associated with a second TURBT, which vary from 1700 to 2900 euros per patient, which could increase substantially if it is associated with complications and requires prolonged hospitalization [52,53,54].

By improving classic techniques such as MTS using deep learning algorithms, we provide a tool that could mitigate inter-observer variability; however, this is an exploratory study, so it serves as a reference for future research aiming to optimize diagnostic accuracy and personalized surgical planning

## 4. Materials and Methods

Study: Retrospective, observational, analytical, and exploratory study. A retrospective, observational, and analytical study was conducted to develop a convolutional neural network (CNN) model capable of differentiating between muscle-invasive (MI) and non-muscle-invasive (NMI) tumors in bladder biopsies stained with Masson’s trichrome technique.

Patients: A retrospective cohort of BC patients from the Dr. Franco Ravera Zunino’s Hospital who underwent TURBT and radical cystectomy between January 2022 and October 2024 was included. A total of 32 patients were considered (11 radical cystectomies and 21 TURBT). Patients with non-urothelial tumors, CIS, or metastatic disease at diagnosis were excluded.

Clinical data: Patient clinical information was obtained from the pathological biopsy reports (T, tumor grade, muscle invasion, specimen weight) and the hospital clinical electronic record system.

This study was approved by the Scientific Ethical Committee of Servicio de Salud Metropolitano Oriente; the waiver of informed consent was approved by the committee.

Tumor samples and Image processing: Biopsies were obtained by TURBT or radical cystectomy procedures and were formalin-embedded and paraffin-fixed (FEPF). All biopsies were analyzed by the same pathologist from the Dr. Franco Ravera Zunino Hospital. From the selected cases, 3–4 µm sections of FEPF samples were obtained and collected on positively charged slides. Sections were stained with Hematoxylin Eosin using the ST Infinity HE Staining System kit 3801698 (Leica Biosystems, Nussloch, Germany) in an automated mode in Autostainer ST5020 equipment (Leica Biosystems, Nussloch, Germany) following the manufacturer’s instructions. Masson’s trichrome staining was then performed using the Trichrome Staining Kit 860-031 (Roche, Tucson, AZ, USA) in an automated process on Ventana BenchMark Special Stains equipment (Ventana Medical Systems, Tucson, AZ, USA) following the manufacturer’s instructions. Paraffin was removed using the automated deparaffinization step. Tissue sections were then incubated in Bouin’s solution to enhance tissue fixation and improve staining brightness. For ~4 µm sections, an extended Bouin’s incubation was applied by adding an additional 28 min to the standard 32 min protocol. The standard staining protocol was performed using the blue chromogen. Nuclear staining was carried out with hematoxylin, using hematoxylin A followed by hematoxylin B for 12 min. Hematoxylin incubation time was adjusted for 24 min. Red staining intensity for the blue protocol (trichrome red) was optimized by incubation at 60 °C for 24 min. Finally, mordanting was performed using both Mordant 1 and Mordant 2 at 37 °C for 12 and 4 min, respectively.

Photographs with a total magnification of 400× were taken with a Leica FLEXACAM C1 camera; 10 to 15 photographs were taken per biopsy (Leica DM2500 microscope, 400× (40× objective and a 10× eyepiece, Leica Microsystems: Wetzlar, Germany). The photographs were taken with a resolution of 3840 × 2160 pixels, and no color normalization was performed.

One slide per biopsy was selected that had several stroma areas without fragmentation artifacts associated with neoplastic urothelium, invasive or non-invasive, and without sectioning or staining artifacts. In biopsies with little stroma per slide and more than one slide, areas from two or more slides were photographed. The criteria used to select the photographed area was randomized based on no staining or cutting fragmentation artifact.

Machine Learning: A total of 702 histopathological images of bladder tumors were collected for this study. The images were annotated by expert pathologists and categorized into two classes: non-muscle-invasive (NMI), which includes stages Ta and T1, and muscle-invasive (MI) tumors. The images were divided into a training set (80%, n = 562 images) and a test set (20%, n = 140 images). To prevent data leakage and ensure an unbiased estimation of model generalization, data partitioning was performed at the patient level. All images from a given patient were assigned exclusively to a single dataset split, such that no patient contributed images to more than one of the training and validation sets. The division was performed to ensure a balanced distribution between classes (MI and NMI). Data preprocessing and augmentation were performed using image generators from the ImageDataGenerator module of the Keras library (v 3.0.5). The applied transformations included shear (shear range = 0.2), zoom (zoom range = 0.2), and random horizontal flip (horizontal flip = True). Images were processed in batches of 3 (batch size = 3) during training and evaluation. A convolutional neural network (CNN) was employed for the classification task. A transfer learning strategy based on the pre-trained VGG16 architecture, initialized with ImageNet weights, was used. The original fully connected layers were excluded, initially freezing the convolutional layers of the pre-trained model to preserve relevant general features. Subsequently, a custom architecture was incorporated, consisting of (a) a fully connected (Dense) layer with 128 neurons and a Rectified Linear Unit (ReLU) activation function, (b) a Dropout layer set to 35% (0.35) to reduce overfitting, and (c) a final classification layer with Sigmoid activation for binary discrimination between MI and NMI (T1 and Ta).

An additional fine-tuning phase was performed, selectively unfreezing the last convolutional layers of the pre-trained model to optimize its performance with our specific images. The model was compiled using the Adam optimizer with an initial learning rate of 0.0001, a binary cross entropy loss function, and the accuracy metric to evaluate the model’s accuracy during training and validation. It was initially trained for 50 epochs. Furthermore, the early stopping technique was employed, monitoring the “validation loss” metric with a patience of 5 epochs to prevent overfitting. To avoid subject-level data leakage and artificially inflated performance estimates, dataset splitting was conducted at the patient level rather than at the image level. Specifically, all images belonging to a single patient were consistently assigned to the same fold during cross-validation. Model performance was assessed using group-based 10-fold cross-validation, with patient identifiers serving as grouping factors, thereby ensuring strict independence between training and validation data. Performance metrics, including accuracy, precision, sensitivity, and specificity, were calculated to comprehensively evaluate the model.

The entire model development and training was implemented using Python version 3.9, in the Spyder 6.0 environment, and Anaconda Navigator software v2.7.0. The TensorFlow 2.10 and Keras libraries were primarily used for model construction and training. Training was performed on a machine equipped with a 10-core GPU and 24 GB of RAM.

Data Analysis: Finally, the model probabilities were correlated with the aforementioned clinical variables of the patients. Statistical analysis was performed with IBM SPSS Statistics v25 using chi square (χ2) or Fisher’s exact test as appropriate for association analyses. Spearman correlation analysis was performed to evaluate correlation between quantitative variables and the probabilities of the model. Statistical significance was set at *p* < 0.05.

**Figure 3:**
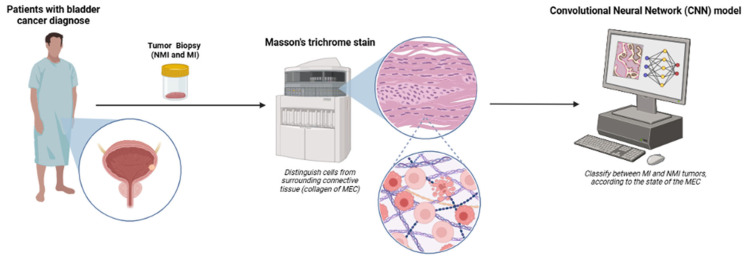
Workflow for CNN-based classification of bladder cancer using Masson’s trichrome-stained biopsies. Tumor biopsies from patients diagnosed with bladder cancer classified as non-muscle-invasive (NMI) or muscle-invasive (MI) were processed using Masson’s trichrome staining to differentiate tumor cells from the surrounding extracellular matrix (ECM), particularly collagen fibers. High-resolution images of stained sections were then used as input for a convolutional neural network (CNN) model and automatically classify tumors as MI or NMI based on microenvironmental features.

Figure 3 graphically summarizes the methodology used.

## 5. Conclusions

In summary, AI has been applied extensively to the bladder and other organ systems, serving as a pivotal tool to enhance diagnostic accuracy when integrated into medical assessments. Our study suggests that CNN-based analysis of MTS histological images facilitates the differentiation of NMIBC from MIBC, thereby reducing inter-observer variability among pathologists. By optimizing the interpretation of the first sample, this approach prevents under- or overtreatment and may preclude the need for secondary TURBT or radical cystectomy in cases where the muscularis mucosae is difficult to distinguish from the muscularis propria.

Such advancements offer significant potential to reduce patient morbidity, mortality, and the overall disease burden. This is particularly valuable in clinical centers lacking subspecialized uropathologists, where diagnostic and staging variability is often higher. While the integration of digital histopathology platforms and diagnostic software requires an initial investment, the long-term reduction in unnecessary surgical interventions provides a clear cost–benefit gain. Ultimately, these findings underscore the utility of MTS not merely as a descriptive technique but as a foundation for quantitative algorithms and a starting point for future research into the relationship between the tumor microenvironment and oncological outcomes.

## Figures and Tables

**Table 1:** Patients’ clinical characteristics. Data is shown as average (± standard deviation) or percentage of total sample, accordingly. ^a^ Mann–Whitney U, ^b^ Test Chi Square or Fisher’s exact test.

	NMIBC	MIBC	*p* Value
Age	73 (±14)	66.38 (±18)	0.35 ^a^
Charlson	5.5 (±3)	4 (3)	0.52 ^a^
BMI	27.6 (±6.1)	29.7 (±12)	0.85 ^a^
Tabaquism	45.45%	40%	0.98 ^b^
Weight (g)	2.5 (±2.6)	11 (±5.5)	0.01 ^a^
Model’s Probability	0.487	0.998	0.001 ^a^

**Table 2:** Diagnostic results of the artificial intelligence model for NMIBC and MIBC diagnosis.

Variable	Value
Accuracy (training)	95.2%
Accuracy (validation)	90.1%
Tumoral grade	*p* = 0.005
Tumoral weight	*p* = 0.017
Tumoral size	*p* = 0.01

**Table 3:** ROC curves of CNN model. PPV: positive predictive value; NPV: negative predictive value.

Variable	Value
AUC	0.8
Sensibility	0.9
Specificity	0.91
PPV	0.82
NPV	0.95

**Table 4:** Multivariate analysis including size, grade weight, and probability. OR: Odds Ratio.

Variable	OR	Confidence Interval	*p* Value
Size	0.940	0.837–1.056	0.296
Grade	0.236	0.003–17.524	0.51
Weight	1.012	0.921–1.113	0.801
Probability	2368.8	3.589–1563659.4	0.019

## Data Availability

The original contributions presented in this study are included in the article. Further inquiries can be directed to the corresponding author.

## Abbreviations

The following abbreviations are used in this manuscript:

BC	Bladder Cancer
NMIBC	Non-Muscle-Invasive Bladder Cancer
MIBC	Muscle-Invasive Bladder Cancer
MTS	Masson’s Trichrome Staining
ECM	Extracellular Matrix
CNN	Convolutional Neural Network
TURBT	Transurethral Resection of Bladder Tumor
RC	Radical Cystectomy
ML	Machine Learning
AI	Artificial Intelligence
MMPs	Matrix Metalloproteinases
FEPF	Formalin-Embedded and Paraffin-Fixed
HE	Hematoxylin Eosin
CIS	Carcinoma in situ
AUC-ROC	Area Under the Receiver Operating Characteristic curve
OR	Odds Ratio
